# Current state of microbiota clinical applications in neonatal and pediatric bacterial infections

**DOI:** 10.1080/19490976.2025.2529400

**Published:** 2025-07-06

**Authors:** Thanh Thien Le, Tram Ngoc Hoang, Dylan Huy Do, Xuan-Hung Nguyen, Cynthia Huynh, Hang Dao Viet, Vu Quoc Dat, Karsten Zengler, Jack A. Gilbert, Sean N. Avedissian, Tri Minh Tran, Jennifer Le

**Affiliations:** a Vinmec-VinUni Institute of Immunology, College of Health Science, VinUniversity, Hanoi, Vietnam; b University of California San Diego Skaggs School of Pharmacy and Pharmaceutical Sciences, La Jolla, CA, USA; c Department of Biological Sciences, Dartmouth College, Hanover, NH, USA; d Tylan Health, El Monte, CA, USA; e Vinmec Hi-Tech Center, Vinmec Healthcare System, Hanoi, Vietnam; f Internal Medicine Faculty, Hanoi Medical University, Hanoi, Vietnam; g Endoscopy Center, Hanoi Medical University Hospital, Hanoi, Vietnam; h Department of Infectious Diseases, Hanoi Medical University, Hanoi, Vietnam; i Department of Pediatrics, University of California San Diego, La Jolla, CA, USA; j Department of Bioengineering, University of California San Diego, La Jolla, CA, USA; k Center for Microbiome Innovation, University of California San Diego, La Jolla, CA, USA; l Program in Materials Science and Engineering, University of California San Diego, La Jolla, CA, USA; m Center for Marine Biotechnology and Biomedicine, Scripps Institution of Oceanography, La Jolla, CA, USA; n Antiviral Pharmacology Laboratory, College of Pharmacy, University of Nebraska Medical Center, Omaha, NE, USA; o Internal Medicine, Kaiser Permanente, Los Angeles, CA, USA; p Center on International Pharmacy Education and Research, University of California San Diego Skaggs School of Pharmacy and Pharmaceutical Sciences, La Jolla, CA, USA

**Keywords:** Microbiome, gut microbiome, nasopharyngeal microbiome, oral microbiome, infection, sepsis, cancer, acute lymphoblastic leukemia, hematopoietic stem cell transplantation, bloodstream infection, *Clostridioides difficile*, antibiotics, probiotics, volatile organic compounds, fecal microbiota transplantation, neonates, pediatrics, children

## Abstract

The microbiota plays a crucial role in pediatric health by shaping immune development and influencing infection susceptibility. In infants and children, an immature microbiota may compromise immune defense, increasing the risk of bacterial infections. This review evaluates clinical trials on the microbiota’s role in neonatal and pediatric bacterial infections, including sepsis, infections in pediatric cancer patients, and *Clostridioides difficile*-associated dysbiosis. We summarized original research articles published from 2000 to May 2024 on the microbiota and bacterial infections in neonates and children. A balanced microbiota is essential for infection prevention, particularly in premature infants and immunocompromised children. Studies of microbiome signatures in the gut, oral cavity, and nasopharynx have highlighted how microbiota composition influences infection risk, treatment response, and adverse effects from antibiotics and chemotherapy. Disruptions from antibiotic exposure, chemotherapy, and hematopoietic stem cell transplantation frequently lead to dysbiosis, characterized by depletion of commensal bacteria and overgrowth of pathobionts, including antibiotic-resistant strains such as *C. difficile*. Conversely, microbiota-restorative interventions, such as probiotics and fecal microbiota transplantation, show promise in reducing bacterial infections by enhancing microbial resilience. The microbiota plays a critical role in predicting and potentially treating bacterial infections in children. While antibiotics remain essential, their widespread use has significant consequences for microbiota health. Striking a balance between effective infection control and microbiota preservation is crucial, particularly in vulnerable pediatric populations. Implementing judicious antibiotic use and exploring microbiota-based therapies may mitigate long-term microbiota disruptions, ultimately improving infection outcomes and overall pediatric health.

## 1. Introduction

The microbiota, a complex community of microorganisms inhabiting the human body, plays a crucial role in maintaining health, particularly during childhood when the microbiota is still developing. From birth, the microbiota undergoes a dynamic maturation process that ultimately achieves a diverse and balanced state. This equilibrium is critical for various physiological processes, including immune system development, digestion, and defense against pathogens. Notably, the microbiota profoundly influences immune system function, shaping susceptibility to infections and modulating responses to microbial threats. A well-balanced microbiota enables the immune system to distinguish between pathobionts and symbionts, fostering an environment that supports health.^1^ In particular, it hinders pathogenic colonization in the gastrointestinal tract – a primary reservoir of microbes capable of causing systemic infections, such as sepsis. Key gut microbiota members, such as those from the phyla *Bacteroidetes* and *Firmicutes*, produce short-chain fatty acids (SCFAs) that regulate gene expression in regulatory T-cells and modulate the antimicrobial activity of macrophages, thereby fortifying the body’s defenses.^2,3^

Conversely, dysbiosis, or an imbalance in gut microbial species, undermines the body’s ability to detect and appropriately respond to pathogens. This disruption heightens the risk of severe infections, including sepsis, bloodstream infections, especially immunocompromised individuals such as preterm infants and pediatric cancer patients.^2,4–10^ Dysbiosis is often triggered by external factors, with antibiotics being a primary driver. Both broad- and narrow-spectrum antibiotics, particularly those with anti-anaerobic activity, can profoundly disrupt microbiota composition. This disruption facilitates the overgrowth of pathogenic species, such as *Enterococcus faecium* and *Klebsiella pneumoniae*, which are normally present in low abundance.^3,8^ Such effects are especially critical in neonates and infants, whose microbiota are still in a fragile state of development.

In this review, we critically evaluate published clinical trials on the microbiota in relation to bacterial infections for at-risk pediatric population such as preterm infants and cancer patients. For each condition, we provide an overview of its clinical burden, pathophysiology, and available microbiome-related data. Furthermore, we examine the far-reaching effects of antibiotics and cancer treatments on the microbiota within this vulnerable population. How these interventions disrupt the microbiota in the gut, oral cavity, and nasopharynx will also be reviewed. By synthesizing current evidence, this review aims to deepen our understanding of the critical role of the microbiota in pediatric infectious diseases and inform approaches to optimize both treatment and microbiota health.

## 2. Methods

**Figure 1.**
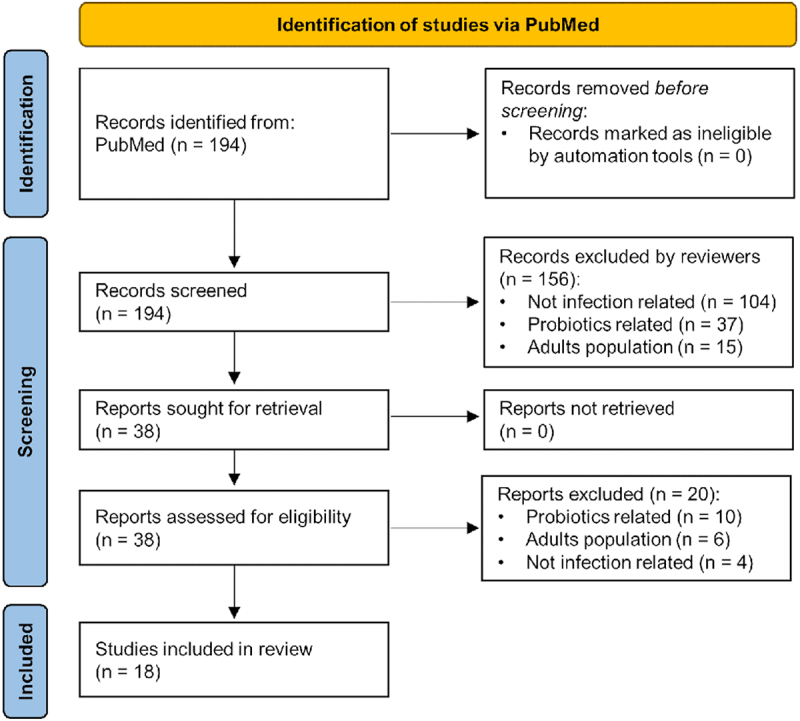
PRISMA flow diagram shows the systematic process we followed to include papers captured by our search.

Original research articles pertaining to the microbiota and different neonatal and pediatric infections were identified and collected using PubMed for period from 2000 to May 2024 following PRISMA guidelines (Figure 1). Search terms such as “microbiome, microbiota, pediatric, children, neonates, sepsis, infection and cancer” were used. Only studies categorized as “clinical trial” and “randomized clinical trial” were included. Clinical trials on probiotics and fecal microbiota transplantation were excluded due to multiple existing systematic reviews, which we summarized.

Three independent reviewers screened the titles and abstracts to identify the articles relevant to the review. Any discrepancies between the reviewers were resolved through discussion to ensure consistent application of the eligibility criteria.

## 3. Results

We identified 194 relevant studies, of which 38 met the inclusion criteria for full-text screening (Figure 1). Following full-text review, 18 original research articles were included in this review (Table 1), comprising 8 studies on sepsis (7 in neonates, 1 in pediatric patients) and 10 studies on infections in pediatric cancer patients.

**Table 1.** Summary of clinical trials on microbiome and clinical outcomes by infection type.

Clinical trials/Design	Country	Population Analyzed	Disease/Condition	Drug/Agent Evaluated	Source of Microbiota	Organism Identified	Clinical Outcomes
SEPSIS							
NEONATES							
Mukhopadhyay S, Lee J-J, Hartman E, et al. **Preterm infants at low risk for early-onset sepsis differ in early fecal microbiome assembly**. Gut Microbes 2022; 14:2154091.^6^ Prospective cohort study	USA	48 infants with very low birth weight (VLBW) ( < 1,500 g) and gestational age < 33 weeks (18 classified as low-risk for EOS (LRE) and 30 classified as non-LRE	Early-onset sepsis	Fecal samples as a marker of gut microbiome changes	Intestinal	*Staphylococcus epidermidis*, Enterobacteriaceae sp.	- LRE infants had higher human-to-bacterial DNA ratios in days 1–3, indicating reduced microbial acquisition. By days 4–7, they showed distinct microbial profiles, with more Staphylococcus epidermidis vs. Enterobacteriaceae in non-LRE. These differences aligned with virulence factor and resistance gene patterns. - Eight of nine late-onset bacteremia cases occurred in non-LRE infants. Pathogens were detectable in fecal samples before bloodstream infection. - Overall, the LRE phenotype is linked with distinct early microbiome features and lower infection risk.
Reyman M, van Houten MA, Watson RL, et al. **Effects of early-life antibiotics on the developing infant gut microbiome and resistome: a randomized trial**. Nat Commun 2022; 13:893.^7^ Randomized, observational, non-blinding, multicenter study	The Netherlands	147 infants with indication for broad-spectrum antibiotic treatment due to suspected sEOS, 80 healthy controls	Early-onset sepsis	Amoxicillin + cefotaxime	Gut	Bifidobacterium	- Broad-spectrum antibiotics for sEOS can have long-term ecological effects.- PCN + gentamicin caused the least microbiome disruption, likely due to limited gut penetration, though its use is constrained by dosing frequency and monitoring needs.- Amoxicillin + cefotaxime most strongly altered microbial composition and resistance gene profiles.- Co-amoxiclav + gentamicin caused the greatest loss in diversity and persistent resistance gene presence.
Morowitz MJ, Katheria AC, Polin RA, et al. **The NICU Antibiotics and Outcomes (NANO) trial: a randomized multicenter clinical trial assessing empiric antibiotics and clinical outcomes in newborn preterm infants**. Trials 2022; 23:428.^5^ Multi-institutional, double-blinded RCT	USA and Canada	802 extremely-low-birthweight (ELBW) infants ( < 1000 g)	Early-onset sepsis	Empiric antibiotics (ampicillin, gentamicin) or placebo	Gut	Pending	−Study under recruitment as of 2023
Berkhout DJC, Niemarkt HJ, Buijck M, et al. **Detection of Sepsis in Preterm Infants by Fecal Volatile Organic Compounds Analysis**. Journal of Pediatric Gastroenterology and Nutrition 2017; 65:e47–e52.^3^ Prospective multi-center study	The Netherlands	248 preterm Infants with gestational age of < 30 weeks	Late-onset sepsis ( > 72 hours after birth)	Fecal samples (from birth until the postnatal age of 28 days)	Intestinal	Firmicutes, Proteobacteria, Clostridium, Klebsiella, Veillonella	-Fecal volatile organic compounds (VOC) profiles of infants LOS could be discriminated from controls by unique set of VOCs that is different from infants without LOS at 3 days, 2 days and 1 day before the onset of LOS - Fecal VOC profiles of preterm infants with LOS could be discriminated from matched controls, up to 3 days before clinical onset of the disease, underlining the hypothesis that intestinal microbiota may play an etiological role in LOS. Notably, VOC profiling is clinically feasible and the potential of this technique in the early detection of LOS needs to be confirmed in future studies
Berkhout DJC, van Keulen BJ, Niemarkt HJ, et al. **Late-onset Sepsis in Preterm Infants Can Be Detected Preclinically by Fecal Volatile Organic Compound Analysis: A Prospective, Multicenter Cohort Study**. Clinical Infectious Diseases **2019**; 68:70–77.^2^ Prospective, multi-center cohort study	The Netherlands and Belgium	843 preterm infants with gestational age ≤30 weeks	Late-onset sepsis	Fecal samples (up to postnatal age of 28 days)	Intestinal	Escherichia coli, Staphylococcus aureus, Staphylococcus epidermidis	- Fecal VOC analysis allowed for preclinical discrimination between infants developing LOS and matched controls. Early detection of LOS may provide clinicians a window of opportunity for timely initiation of individualized therapeutic strategies aimed at prevention of sepsis, possibly improving LOS-related morbidity and mortality - LOS caused by E.coli, S. aureus and S. epidermidis could be differentiated from their matched controls with high predictive value, up to 3 days before clinical onset of LOS
Thatrimontrichai A, Surachat K, Singkhamanan K, Thongsuksai P. **Long Duration of Oral Care Using Mother’s Own Milk Influences Oral Microbiota and Clinical Outcomes in Very-low-birthweight Infants: Randomized Controlled Trial**. The Pediatric Infectious Disease Journal 2023; 42:804.^9^ Open label RCT	Thailand	116 VLBW (birthweight of ≤1500 g) neonates with gestational age between 22 weeks, 0 days and 31 weeks, 6 days	Necrotizing enterocolitis (NEC), late-onset sepsis (LOS), ventilator-associated pneumonia (VAP)	Mother’s own milk (MOM)	Oral, Gut	Bifidobacterium bifidum, Faecalibacterium, Pseudomonas	- Prolonged (22-day) oral care with MOM in VLBW infants maintained healthy bacteria like B. bifidum and Faecalibacterium. - MOM significantly reduced clinical sepsis risk vs. sterile water (47% vs. 76%; NNT = 4), without affecting mortality or other severe outcomes.
Sanyang B, de Silva TI, Kanteh A, et al. **Effect of intra-partum azithromycin on the development of the infant nasopharyngeal microbiota: A post hoc analysis of a double-blind randomized trial**. eBioMedicine 2022; 83:104227.^8^ Double-blind randomized placebo-controlled clinical trial	The Gambia	109 healthy infants (Birth to 12 months)	Sepsis	Azithromycin	Nasopharyngeal	Staphylococcus spp., Moraxella spp.	- Intrapartum azithromycin increased microbial diversity at day 6 and changed overall composition at days 6 and 28. It reduced Staphylococcus (day 6) and increased Moraxella (12 months). - This reduction in Staphylococcus, a major neonatal sepsis pathogen, may be beneficial. Moraxella is associated with a healthy nasopharynx. - The short-term microbiome changes warrant further study to assess long-term effects.
PEDIATRICS							
Liu J, Wang M, Chen W, et al. **Altered Gut Microbiota Taxonomic Compositions of Patients With Sepsis in a Pediatric Intensive Care Unit**. Frontiers in Pediatrics 2021; 9.^4^ Single-center case control study	China	20 children with sepsis in pediatric intensive care unit (PICU) and 20 healthy children (ages 1–8)	Sepsis	Fecal samples	Gut	Enterococcaceae, *Enterococcus*, *Enterococcus durans*, Bifidobacteriales	− Children with sepsis had significantly lower gut microbiota diversity than healthy peers. - Enterococcaceae, including Enterococcus and E. durans, were elevated and positively correlated with CRP and WBC. - Acidaminococcaceae and Bifidobacteriales were reduced, with Bifidobacteriales inversely correlated with inflammation (WBC).
CANCER INFECTIONS – PEDIATRICS							
Lähteenmäki K, Wacklin P, Taskinen M, et al. **Haematopoietic stem cell transplantation induces severe dysbiosis in intestinal microbiota of pediatric ALL patients**. Bone Marrow Transplant 2017; 52:1479–1482.^30^ Longitudinal, observational study	Finland	5 pediatric ALL patients treated with allo-HSCT, 6 ALL patients treated with chemotherapy	Graft vs host disease (GvHD)	Fecal samples	Gut	**Diseased**: *Enterococcus, Staphylococcus, Enterobacter, Bacteroides,Lachnospriraceae* **Healthy**: *Bifidobacterium, Bacteroides, Blautia* and *Faecalibacterium, Roseburia*	- Reduced diversity: chemotherapy patients have lower inverse Simpson diversity index, while allo-HSCT patients have even lower diversity - Bifidobacterium, Bacteroides, Blautia and Faecalibacterium, Roseburia are dominant in healthy donor - Enterococcus, Staphylococcus, Enterobacter, Bacteroides,Lachnospriraceae constitute 90% of HSCT patients microbiota - Patient microbiota gradually revert to the healthy state after treatment (higher diversity, more Blautia, Bifidobacterium and Roseburia) - Microbiota of patients with severe GvHD is dominated by Proteobacteria, while the microbiota of patients with no GvHD stabilize to normal at a faster rate.
Severyn CJ, Siranosian BA, Kong ST-J, et al. **Microbiota dynamics in a randomized trial of gut decontamination during allogeneic hematopoietic cell transplantation**. JCI Insight 2022; 7: e154344. Placebo-controlled RCT	USA	20 pediatric patients undergoing HCT	Blood stream infection (BSI)+A26:H27	Prophylaxis oral vancomycin-polymyxin B	Gut microbiome	Staphylococcus	- No significance difference between Shannon diversity, GvHD, and immune profile between treated and non-treated group − 8 episodes of BSI in the non-treated group compared to 1 episode in the treated group - Prophylaxis oral vancomycin-polymyxin B reduce the presence of pathobionts in the gut to potentially prevent gut-derived BSI.
Badia P, Andersen H, Haslam D, et al. **Improving Oral Health and Modulating the Oral Microbiome to Reduce Bloodstream Infections from Oral Organisms in Pediatric and Young Adult Hematopoietic Stem Cell Transplantation Recipients: A Randomized Controlled Trial**. Biol Blood Marrow Transplant 2020;26(9):1704–1710.^40^ Open-label single-center 1:1 RCT	USA	35 pediatric hematopoietic stem cell transplant recipients age > 2 years	Bloodstream infection (BSI)	Oral sample collection for microbiome analysis	Oral	*S. mitis, Gamella haemolysans, Fusobacterium periodonticym, Streptococcus gordonii, E. coli, K. pneumoniae, Serratia marcescens, Bacteroides vulgaris, C. albicans, Malassezia restricta, S. aureus*, *Streptococcus* spp	- Healthy children and young adults had significantly greater oral microbiome diversity and commensal species abundance (e.g., S. mitis, Gamella haemolysans, F. periodonticym, S. gordonii) compared to HSCT recipients. In contrast, HSCT recipients had elevated levels of potential pathogens like E. coli, K. pneumoniae, S. marcescens, and C. albicans. - At baseline, microbiomes of SOC and xylitol-treated HSCT patients were similar. However, by day 7, the xylitol group showed reduced abundance of pathogens including S. aureus (*p* = .036) and K. pneumoniae (*p* = .022), suggesting xylitol may mitigate oral dysbiosis post-transplant.
van de Velde ME, El Hassani SEM, Kaspers GJL, et al. **Prediction of Bloodstream Infection in Pediatric Acute Leukemia by Microbiota and Volatile Organic Compounds Analysis**. J Pediatr Hematol Oncol 2022; 44: e152–e159.^36 −^ Single-center case-control pilot study	The Netherlands	6 pediatric acute leukemia patients (ages 3–20)	Bloodstream infection (BSI)	Fecal samples	Intestinal	*Bacteriodetes*, *Pantoea spp.*	-In total, 6 patients were included, of which 1 developed BSI and 1 neutropenic fever. Both showed reduced microbial diversity and stability of *Bacteroidetes*. In the BSI case, *Pantoea* was identified 15 days before BSI. Significant differences in fecal VOC profiles were measured between the case and controls
Mendonça RMH de, Araújo M de, Levy CE, et al. **Oral Mucositis in Pediatric Acute Lymphoblastic Leukemia Patients: Evaluation of Microbiological and Hematological Factors**. Pediatr Hematol Oncol 2015; 32:322–330.^37^ Single-center, longitudinal, observational study	Brazil	71 pediatric ALL patients	Oral mucositis	Oral samples	Oral	Candida spp.	- On Day 14 of Brazilian Childhood Cooperative Group-protocol ALL-99 (GBTLI ALL-99), there is a significant association between *Candida spp.*, platelet count, HSV-1 and severity of oral mucositis. - On Day 56, severity of mucositis is associated with HSV-1 and neutrophil count
Hakim H, Dallas R, Wolf J, et al. **Gut Microbiome Composition Predicts Infection Risk During Chemotherapy in Children With Acute Lymphoblastic Leukemia**. Clin Infect Dis 2018; 67:541–548.^34^ Single-center, longitudinal, observational study	USA	199 newly diagnosed pediatric ALL patients	Febrile neutropenia, Diarrhea	Fecal samples	Gut	Bacteroidetes, Clostridiaceae and Streptococcaceae, Proteobacteria, Enterococcaceae	- Microbial diversity (Shannon index) reduced after chemotherapy induction, only reverting to baseline level after less intensive regiments. - Chemotherapy induced the decrease of Bacteroidetes, Faecalibacterium, Ruminococcaceae, Actinobacteria, Verrucomicrobia; and the increase of Clostridiaceae, Streptococcaceae, Lactobacillaceae, Enterococcaceae, and other Firmicutes in patients gut microbiota. - Patients with Proteobacteria RA ≥ 0.01% have a 67% chance of developing febrile neutropenia.- Enterococcaceae dominance is associated with neutropenia and Streptococcaceae dominance is associated with diarrhea.
Wada M, Nagata S, Saito M, et al. **Effects of the enteral administration of Bifidobacterium breve on patients undergoing chemotherapy for pediatric malignancies**. Support Care Cancer 2010; 18:751–759.^42^ Placebo-controlled RCT	Japan	42 patients with pediatric malignancies undergoing chemotherapy (ages 1–13)	Fever, diarrhea, antibiotic usage	Fecal samples	Gut	Bifidobacterium breve strain Yakult, Enterobacteriaceae, Bacillus, Enterococcus	- Various cancer types: ALL, non-Hodgkin lymphoma, yolk sac tumor, Ewing sarcoma, acute myeloid leukemia, Hodgkin disease, primitive neuroectodermal tumor, leiomyosarcoma) - The frequency and duration of febrile episodes was less in the probiotic group than in the placebo group: fever happens in 44% in the probiotic group and 68% in the placebo group.- Probiotics inhibit the growth of facultative anaerobes such as Enterococcus and Enterobacteriaceae.- There was no significant intergroup difference in the frequency and duration of diarrhea, use of antibiotics, NK and WBC count, use of G-CSF, maximum value of C-reactive protein (CRP), number of CRP elevated episodes or concentration of total organic acid and short fatty chain acids.
Eghbali A, Ghaffari K, Khalilpour A, Afzal RR, Eghbali A, Ghasemi A. **The effects of LactoCare synbiotic administration on chemotherapy-induced nausea, vomiting, diarrhea, and constipation in children with ALL: A double-blind randomized clinical trial**. Pediatr Blood Cancer 2023; 70:e30328.^43^ Double-blind, placebo-controlled RCT	Iran	113 pediatric ALL patients (58 treated, 55 placebo) (ages 5–15)	Chemotherapy-induced diarrhea, nausea, vomiting, and constipation	LactoCare	Gut	Bacillus clausi, Bacillus subtillis, Lactobacillus sporogenes	- Chemotherapy-induced diarrhea is present in the LactoCare group at 3.7%, 1.8%, and 0% at Day 1, 2, and 3–7, respectively. In the placebo group, diarrhea is present in 11.5%, 13.5%, 11.5% and < 10% at Day 2, 3, 4 and 5–7, respectively.
Ladas EJ, Bhatia M, Chen L, et al. **The safety and feasibility of probiotics in children and adolescents undergoing hematopoietic cell transplantation**. Bone Marrow Transplant 2016; 51:262–266.^41^ Interventional trial	USA	31 pediatric patients undergoing hematopoietic cell transplantation (HCT) −12 having leukemia and 1 having lymphoma (Ages 2–17)	Lactobacillus plantarum bacteremia.	Lactobacillus plantarum probiotics		Lactobacillus plantarum	− No Lactobacillus plantarum bacteremia or adverse events related to Lactobacillus plantarum bacteremia were observed
Tunyapanit W, Chelae S, Laoprasopwattana K. **Does ciprofloxacin prophylaxis during chemotherapy induce intestinal microflora resistance to ceftazidime in children with cancer?** J Infect Chemother 2018; 24:358–362. Placebo-controlled RCT	Thailand	87 pediatric ALL or lymphoma patients (44 treated, 43 placebo) (Ages < 18)	Antimicrobial susceptibility	Ciprofloxacin, ceftazidime	Rectal swabs	Escherichia coli, Klebsiella pneumoniae	- No change in the susceptibility to ceftazidime between treated and non-treated groups. - There is a significant increase in the MIC50 of ceftazidime in the treated group overtime (from 1–3 week after ciprofloxacin treatment)

Abbreviations: ALL, acute lymphocytic leukemia; BSI, bloodstream infection; CRP, C-reactive protein; ELBW, extremely low birthweight; EOS, early-onset sepsis; G-CSF, granulocyte colony-stimulating factor; GvHD, graft vs host disease; HCT, hematopoietic cell transplantation; HSCT, hematopoietic stem cell transplantation; HSV, herpes simplex virus; LOS, late-onset sepsis; LRE, low-risk for early-onset sepsis; MIC, minimum inhibitory concentration; MOM, mother’s own milk; NEC, necrotizing enterocolitis; NICU, neonatal intensive care unit; PICU, pediatric intensive care unit; PCN, penicillin; RA, relative abundance; RCT, randomized controlled trial; sEOS, suspected early-onset sepsis; VAP, ventilator-associated pneumonia; VLBW, very low birthweight; VOC, volatile organic compounds; WBC, white blood count.

Data extraction was performed by three reviewers and consolidated into a structured table (Table 1). The extracted data included study characteristics (e.g. author, year, country), final analyzed population demographics, disease/condition, source of microbiota, organisms identified, and clinical outcomes. This systemic approach ensured the uniform collection and organization of relevant data for subsequent analysis.

## 3. Infections in neonate and pediatric patients

### 3.1. Sepsis

Sepsis is a life-threatening organ dysfunction caused by a dysregulated host response to infection and is a major cause of morbidity and mortality in the neonatal and pediatric populations.^11^ Globally, it was reported that 1.3 million cases of neonatal sepsis occur annually, with a mortality rate of 17.6%.^12–14^ Neonatal sepsis is classified into: early-onset sepsis (EOS) and late-onset sepsis (LOS). EOS usually occurs during the first 72 hours of life, often resulting from vertical transmission of pathogens from mother to baby, while LOS develops at or after 72 hours of life, commonly due to horizontal transmission from the environment or caregivers to the newborn.^12^ Data from 2016 reported approximately 7.5 million sepsis-related deaths annually worldwide.^15^ In the United States, 720,000 children were hospitalized for sepsis, with a mortality rate of 25% and an estimated cost of $4.8 billion.

The relationship between gut microbiota and sepsis is reciprocal, as each can influence the other. Unlike the relatively stable and diverse microbial community in adults, the neonatal gut microbiome is initially dominated by facultative anaerobes and evolves rapidly during early life.^16^ This immature microbiota lacks protective commensals and barrier functions necessary to prevent pathogen colonization, and has been identified as a critical risk factor for sepsis.^4^ During sepsis, neonates often display transient microbial instability with increases in pathobionts such as *Escherichia coli, Klebsiella pneumoniae, Staphylococcus aureus, and Enterococcus species*, alongside reductions in protective genera such as *Bifidobacterium* and *Bacteroides*.^17^ In contrast, adult sepsis is typically characterized by a sharp decline in diversity and dominance by resistant pathogens like *Enterococcus* and *Clostridium difficile*.^18^ Dysbiosis, an imbalance in the host-microbiota equilibrium, can compromise the intestinal barrier by increasing membrane permeability and disrupting immune homeostasis, creating conditions that facilitate bacterial translocation and increase susceptibility to sepsis. Conversely, sepsis itself can exacerbate dysbiosis through hypoxic injury, inflammation, and epithelial damage, often leading to reduced microbial diversity and dominance of pathogenic genera such as *E. coli* or *S. aureus*.^4^ These age-specific patterns of dysbiosis underscore the greater vulnerability of the neonatal microbiome to sepsis-related disruption, with implications for immune development and clinical outcomes. Understanding these differences is essential for designing effective, age-appropriate microbiota-targeted interventions in sepsis management.

While antibiotics have significantly reduced mortality and morbidity associated with bacterial infections, their non-selectivity can worsen the relationship between dysbiosis and sepsis. Broad-spectrum antibiotics disrupt the microbiota, suppressing or eliminating protective bacteria and allowing pathogens to dominate. This disruption not only exacerbates dysbiosis but also increases the risk of secondary infections, compounding the likelihood of sepsis in neonates and pediatric patients.^19^ Additionally, invasive medical devices, such as intravascular catheters, endotracheal tubes, and feeding tubes, provide entry points for bacteria into the body, further increasing the risk of infection. Given the developmental and physiological differences between neonates and older children, the roles of gut, oral, and nasopharyngeal microbiota in sepsis are addressed separately for each population.

#### 3.1.1. Sepsis in neonates

Neonates born preterm ( < 37 weeks gestation), with very low birth weight (VLBW, < 1500 grams), or extremely low birth weight (ELBW, < 1000 grams) are particularly vulnerable to sepsis.^20^ The underdeveloped skin barrier and immature immune system in these infants offer limited protection against pathogenic infection. In addition, increased exposure to broad-spectrum antibiotics and the use of invasive medical devices further exacerbates their risk for infection during their stay in the neonatal intensive care unit (NICU).^12^ Thus, eight out of nine published clinical trials on the relationship between microbiota and sepsis specifically focus on neonates.

##### 3.1.1.1. Gut microbiome and early-onset sepsis

The high prevalence of risk factors for EOS, combined with the overlapping signs between EOS and the physiological immaturity of preterm infants, poses significant challenges in promptly identifying EOS after birth. Consequently, around 80–90% of VLBW infants received empiric antibiotics after birth to ‘rule out’ EOS, despite confirmation in only 1–2% of these cases.^7^ To address this issue, Mukhopadhyay et al investigated the difference between the early intestinal microbiome in preterm infants at low-risk and at-risk for EOS to help prevent the unnecessary use of empiric antibiotics (Table 1).^6^ Low-risk EOS infants were defined by specific criteria: (1) delivery via cesarean section, (2) absence of spontaneous or induced labor, (3) membrane rupture occurring only at delivery, and (4) non-infectious medical reasons at birth, such as maternal preeclampsia or fetal growth restriction. These infants exhibited reduced maternal-to-infant bacterial transmission, potentially eliminating the need for routine antibiotics. In contrast, infants at-risk for EOS received antibiotics within the first 24 hours after birth. Mukhopadhyay’s study revealed distinct microbiome compositions between these groups. Infants at low risk for EOS exhibited a higher ratio of human-to-bacterial DNA in fecal samples during the first 3 days of life, indicating reduced bacterial acquisition compared to their at-risk counterparts. During days 4–7 of life, differences in microbiome composition became evident, with low-risk infants predominantly colonized by *Staphylococcus epidermidis*, whereas at-risk infants predominantly harbored *Escherichia coli*.^7^ Thus, profiling the intestinal microbiome composition of infants could be useful in differentiating low-risk EOS infants from at-risk EOS infants, reducing unnecessary antibiotic exposure and its associated risks.

Up to 10% of neonates with EOS receive broad-spectrum antibiotics,^21^ often in combinations such as amoxicillin or ampicillin with gentamicin or cefotaxime.^8,22–24^ These regimens effectively target the most prevalent pathogens associated with EOS, including group B *Streptococcus, Listeria monocytogenes*, and *E. coli*, but they also carry ecological consequences. For instance, findings from a Dutch NICU study revealed that intravenous amoxicillin resulted in the proliferation of extended-spectrum beta-lactamase (ESBL)-producing bacteria, such as *Klebsiella* spp.^8^ Similarly, administration of 3rd generation cephalosporins, like cefotaxime, was linked to the emergence of resistant *Enterobacter spp*. strains.

A retrospective study by Le et al examined the impact of switching empiric antibiotic therapy from cefotaxime and vancomycin to tobramycin and vancomycin in the NICU at Miller Children’s Hospital, prompted by a rise in infections caused by ESBL-producing bacteria.^25^ Among 250 infants studied, those treated with cefotaxime and vancomycin had a significantly higher incidence of ESBL infections (7.8%) compared to those receiving tobramycin and vancomycin (0.8%, *p* = 0.008). Multivariate analysis revealed that prior exposure to ampicillin and gentamicin, and cefotaxime and vancomycin regimen were both associated with an increased risk of developing ESBL infections, highlighting the importance of antibiotic stewardship in managing neonatal infections.

In a multicenter randomized trial involving 146 neonates suspected of EOS and 80 healthy neonates, Reyman et al investigated the impact of three commonly used antibiotic regimens in the Netherlands: penicillin + gentamicin, amoxicillin/clavulanic acid + gentamicin, and amoxicillin + cefotaxime (Table 1).^8^ The study aimed to identify the optimal regimen for minimizing gut microbiota disruption and reducing the selection of antimicrobial resistance genes. All three regimens reduced microbial diversity, although to varying degrees. Amoxicillin/clavulanic acid + gentamicin caused the most significant decrease in alpha diversity (median Shannon Diversity 0.43), whereas penicillin + gentamicin (median Shannon Diversity 0.75, *p* < 0.001) and amoxicillin + cefotaxime (median Shannon Diversity 0.6) showed less pronounced effects. Microbial colonization patterns were also altered. The proportion of *E. coli* was reduced in the penicillin + gentamicin and amoxicillin + cefotaxime groups but remained unchanged in the amoxicillin/clavulanic acid + gentamicin group. *Akkermansia* species, linked to metabolic regulation, were reduced exclusively in the amoxicillin + cefotaxime group. Meanwhile, *Klebsiella* proportions increased in the penicillin + gentamicin and amoxicillin/clavulanic acid + gentamicin groups during early life but were comparatively lower in these groups than in the amoxicillin + cefotaxime group in later infancy. Additionally, *Acinetobacter* species increased in the amoxicillin + cefotaxime and penicillin + gentamicin groups but not in the amoxicillin/clavulanic acid + gentamicin group.

The regimens also varied in their impact on antimicrobial resistance genes. The amoxicillin + cefotaxime group demonstrated the greatest selection pressure, with a higher number of antimicrobial resistance genes (R^2^ = 11.1%, p.adj = 0.002) compared to amoxicillin/clavulanic acid + gentamicin (R^2^ = 6.3%) and penicillin + gentamicin (R^2^ = 5.9%). These findings suggest that penicillin + gentamicin is the preferred combination, as it resulted in the least ecological disruption, including less microbial diversity reduction and fewer resistance genes. The limited gut lumen penetration of intravenously administered aminoglycosides likely contributed to these favorable outcomes. However, challenges such as frequent penicillin administration and the need to monitor gentamicin serum levels may limit its use.

It should be noted that across all regimens, Antibiotic use had persistent impacts on neonatal gut microbiota, detectable up to 12 months after treatment. Beneficial *Bifidobacterium* species, critical for digesting human milk oligosaccharides and supporting infant growth and immunity, were significantly reduced. Continued breastfeeding failed to restore their levels, likely due to the near-complete elimination of these bacteria caused by early antibiotic exposure. Such changes may have long-term implications for infant health and development.

Further evidence is anticipated from an ongoing large randomized clinical trial by Morowitz et al., involving 802 ELBW infants. This trial aims to evaluate the impact of empiric antibiotic therapy (ampicillin + gentamicin) on the gut microbiome and its potential role in EOS prevention. Investigators hypothesize that fecal samples collected during the first month of life from antibiotic-treated infants will exhibit reduced microbial diversity, an increased prevalence of pathogenic organisms, and diminished commensal anaerobes compared to untreated controls receiving saline.

##### 3.1.1.2. Gut microbiome and late-onset sepsis

Reduced microbial diversity, a hallmark of gut dysbiosis, is a significant risk factor for LOS. Infants at risk for LOS exhibit reduced gut microbial diversity even before the onset of sepsis compared to those without LOS. Notably, *Bifidobacteria*, beneficial bacteria known for their anti-inflammatory effects, were observed to be more than ten-fold higher in infants without LOS.^5,26^

Fecal volatile organic compounds (VOCs) provide a noninvasive means to assess gut dysbiosis and predict LOS at an early, preclinical stage. VOCs are carbon-based substances that can vaporize into the air as gases and can originate from many different sources, such as biological processes and human activities. In the context of microbiome, VOCs are generated by microbial metabolic processes and host metabolism and thus, they offer valuable insights into gut microbial metabolism. Analytical techniques like gas chromatography-mass spectrometry and handheld electronic nose (eNose) devices enable the identification of these compounds.^5^

In a large prospective, multicenter cohort study by Berkhout et al., fecal VOC analysis of 843 premature infants ≤30 weeks gestational age during their first month of life. Intestinal microbial changes were detected up to 3 days before the clinical onset of LOS (Table 1). Infants with LOS caused by a specific pathogen, such as *E.coli, S.epidermidis*, or *S.capitis*, displayed a unique set of VOCs that were different from both those without LOS and other bacterial causes of LOS.^2,4^ Early identification of LOS offers clinicians a window to implement preventive strategies before LOS manifests, potentially enhancing outcomes and reducing morbidity and mortality.

While early detection of gut dysbiosis is crucial, interventions to restore or maintain a healthy microbiome may also reduce LOS risk. A randomized controlled trial by Thatrimontrichai et al. examined the long-term effects of mother’s own milk (MOM) on clinical outcomes in very low birth weight (VLBW) neonates requiring gastric or endotracheal feeding during their first weeks of life (Table 1).^10^ MOM was hypothesized to enhance immune function and protect against infections, including LOS. Although there were no significant differences in LOS, ventilator-associated pneumonia, or necrotizing enterocolitis between the MOM and control groups, MOM significantly reduced the incidence of clinical sepsis (47% vs. 76%; 95% CI, −0.49 to −0.05). MOM’s effects were attributed to its ability to boost salivary and urinary immune-protective factors while reducing urinary pro-inflammatory cytokines. This enhanced immune protection helped maintain a protective physical barrier in the oral cavity, preventing mucosal breakdown, reducing bacterial translocation, and fostering the growth of beneficial microbiota. These benefits suggest that long-term oral care with MOM, sustained over 22 days in the study, played a pivotal role in lowering the incidence of clinical sepsis. The findings underscore the importance of MOM not only as optimal nutrition but also as a critical component of neonatal infection prevention strategies.

##### 3.1.1.3. Oral and nasopharyngeal microbiome

In the initial weeks of life, VLBW neonates are unable to independently feed and breathe, requiring either gastric or endotracheal intubation. While these interventions are necessary, they pose potential risk of infection for neonates by providing pathways for pathogens entry and disrupting the natural balance of the oral microbiota. Breastmilk, the optimal nourishment for infants, contains a range of bioactive proteins and biological substances that regulate the immune response. Furthermore, breastmilk contains prebiotics that can potentially foster the growth of beneficial microorganisms. Thus, oral care with breastmilk during this period can help protect against mucosa cracks and bacterial translocation, reducing the risks of sepsis. The same study by Thatrimontrichai et al. also evaluated the effects of MOM compared to sterile water on oral microbial diversity in VLBW neonates (Table 1).^10^ Although the overall microbial diversity did not differ significantly between the two groups over a median duration of 22 days for MOM and 27 days for sterile water, specific health-promoting bacterial taxa were better preserved in the MOM group. *B. bifidum* and *Faecalibacterium sp*, known for their anti-inflammatory properties, were minimally reduced over time in the MOM group (*B. bifidum* 3.9% to 2.5%, *Faecalibacterium* 8.1% to 6.2%), compared to substantial decrease in the sterile water group (*B. bifidum* 5.5% to 0.9%, *Faecalibacterium* 12.4% to 2.1%).^10^ These findings underscore the importance of MOM in supporting a healthy oral microbiome and preventing pathogenic shifts that could contribute to neonatal infections.

Respiratory tract infections are another source for the development of sepsis.^27^ The nasopharyngeal microbiota complements the oral microbiota as a key defense against airborne pathogens, with the nasopharyngeal mucosa acting as the first barrier. A balanced nasopharyngeal microbiota, together with host innate immunity, suppress opportunistic pathogens such as *Haemophilus influenzae*, *Staphylococcus aureus*, and *Streptococcus pneumoniae*, which are commonly implicated found in the nasopharynx.^28^ Sanyang et al. explored the effect of intrapartum antibiotics on nasopharyngeal microbiota development in a randomized, placebo-controlled trial conducted in sub-Saharan Africa, a region with a high burden of neonatal sepsis caused by *S. aureus* (Table 1).^9^ A single dose of 2 g intrapartum azithromycin or placebo was given prophylactically to mothers 2 hours before delivery, and the neonatal microbiota diversity and composition were evaluated from birth up to 12 months of age. On day 6 after drug administration, azithromycin reduced the prevalence of *S. aureus* (49.08% vs. 66.27%, q = 0.030) compared to placebo, leading to a more even distribution of bacterial species and significantly greater alpha-diversity (mean Shannon index azithromycin 1.86 vs placebo 1.41, *p* = 0.018).^9^ However, by day 28, this difference in alpha-diversity has resolved, likely due to decreasing exposure to azithromycin through breastmilk over time. By month 12, microbiota composition and diversity were largely similar between the azithromycin and placebo arms. However, *Moraxella* (excluding *M. catarrhalis*), a key commensal genus of the infant nasopharynx that has been associated with a healthy nasopharyngeal microbiota, was significantly more prevalent in the azithromycin group (40% vs 29%, *p* = 0.044).^9^

These findings highlight the potential of intrapartum azithromycin to mitigate early-life infection risks. The regiment induced short-term (approximately 6 days) changes in the nasopharyngeal microbiota, inhibiting the excessive growth of key pathogenic Gram-positive *S. aureus* during this critical period. This intervention is particularly valuable in low- and middle-income countries, where neonatal exposure to pathogens and the burden of neonatal sepsis are high.

#### 3.1.2. Sepsis in Pediatrics

Similarly to neonates, alterations of the gut microbiota occurred in older infants and children with sepsis. A study by Liu et al. demonstrated significant dysbiosis in pediatric patients with sepsis compared to their healthy counterparts (Table 1).^2^ Children with sepsis had an increased proportion of opportunistic pathogenic bacteria, substantial depletion of commensal taxa, and a notable reduction in microbial diversity compared to those without. Such imbalances were closely associated with systemic inflammation. For instance, the *Enterococcaceae* family, known for its association with nosocomial infections, can translocate into the bloodstream, leading to bacteremia and sepsis.^4^ The increased abundance of *Enterococcaceae*, such as *Enterococcus durans*, in the GI tract of septic children correlated positively with elevated inflammatory markers such as C-reactive protein and white blood cell counts.^2^ In contrast, *Acidaminoccaceae* members, known for producing short-chain fatty acids (SCFAs), are vital for T-cell differentiation and mucosal immune development. Similarly, *Bifidobacteriales* are essential for regulating gastrointestinal immune responses and maintaining overall gut health.^4^ Indeed, a decreased abundance of *Acidaminoccaceae* and *Bifidobacterales* resulted in a decreased WBC count.^2^ This evidence further underscores the importance of the gut microbiota in pediatric sepsis and the potential value of therapies targeting microbial balance

The body of research reviewed in this section highlights the critical relationship between the microbiome and immune function in neonatal and pediatric populations, particularly in the context of sepsis. Across all age groups, a consistent pattern of microbial dysbiosis emerges: a decline in microbial diversity, loss of beneficial taxa, and overgrowth of harmful bacteria. These disruptions not only reflect the immune system’s dysfunction but also exacerbate the severity of infections by promoting systemic inflammation and weakening mucosal barriers.

### 3.2. Clostridioides difficile infection

One of the most well-characterized examples of microbiota-driven infection is *Clostridioides difficile* infection (CDI), which underscores the consequences of disrupted microbial balance in the pediatric gut. CDI is primarily caused by disruption of the gut microbiota, often following antibiotic use, allowing overgrowth of toxigenic *C. difficile* strains. These strains produce toxins A and B, which disrupt the colonic epithelial barrier, leading to inflammation, diarrhea, and colitis. CDI in pediatric populations exhibits unique epidemiological patterns, with high rates of asymptomatic colonization in infants and rising rates of symptomatic infection in older children.^29,30^ Colonization rates among infants can reach up to 70%, attributed to immature gut microbiota and lack of toxin receptor expression.^31^ In older children, CDI is increasingly observed, particularly in healthcare-associated settings, but also in community-acquired cases.^32^

Previous review articles have highlighted the role of gut dysbiosis in CDI among neonates and pediatric populations.^33,34^ Infants and children with exposure to antibiotics experienced a delay in colonization of *C. difficile* and other fecal microbiota. However, this decrease is only present during antibiotic treatment, and *C. difficile* colonization quickly resumed within 2 to 4 weeks following antibiotic discontinuation.^35^ Both genetic and culture-based studies demonstrated a notable reduction in gut microbiota diversity and resilience during CDI, in which there was a decline in *Firmicutes, Bacteroidetes*, and *Actinobacteria*, and an increase in *Proteobacteria*.^33^ These microbial imbalances lead to reduced production of butyrate, a critical short-chain fatty acid, and elevated levels of lactic acid bacteria, further contributing to the inflammatory state.

In infants, the high prevalence of asymptomatic *C. difficile* colonization is thought to result from several protective factors. The immature intestinal environment, characterized by a less diverse microbiota, is dominated by beneficial bacteria such as *Bifidobacteria*, which promote localized anti-inflammatory responses. Additionally, a high abundance of *Ruminococcus* species appears to mitigate the adverse effects of *C. difficile* toxins. Additionally, infants lack mature receptors for clostridial toxins, and they benefit from immunological factors including toxin-neutralizing antibodies. Together, these factors contribute to the absence of clinical symptoms despite colonization.^33^ However, further research is needed to better understand whether specific composition of gut microbiota contributes to either asymptomatic carriage or clinical infection by *C. difficile*. Moreover, the mechanisms involved in the interaction between *C. difficile*, commensal gut bacteria, and specific compounds within the gut are not fully understood. Nevertheless, adjunctive therapies targeting the microbiota, such as probiotics, prebiotics, postbiotics, synbiotics, non-toxigenic bacteria, or FMT, have demonstrated significant efficacy in managing CDI.^33,36^ According to the 2023 WGO guidelines for probiotics and prebiotics, 250–500 mg of probiotic strain *Saccharomyces boulardii* is effective in reducing risk of *C. difficile* associated diarrhea in the pediatric population.^36^ Interestingly, FDA has approved two microbiota-based treatments for the prevention of recurrence of *C. difficile* infections in adults, REBYOTA ^TM^ (Ferring Pharmaceuticals Inc.) and VOWST ^TM^ (Seres Therapeutics, Inc.).^37,38^ Although the mechanism is not yet fully established, the products aimed to restore the balance of the gut microbiota, increase the production of secondary bile acids, and thereby reduce the germination of *C. difficile* spores. This again implies the important role of gut microbiota in *C. difficile* infection.

### 3.3. Probiotics and fecal matter transplantation

Antibiotic-induced dysbiosis and the emergence of multi-drug resistant organisms such as CDI pose significant challenges in neonates and children. These concerns have encouraged active research into alternative antimicrobial strategies, notably probiotics and FMT. Numerous reviews have summarized the current consensus on these interventions in pediatric infections, which we briefly presented here.^39–49^

Both probiotics and FMT aim to restore microbial balance by introducing exogenous microbes into the gastrointestinal tract. The goal is to promote colonization by beneficial microbes, displace pathogenic bacteria, and reestablish homeostasis. Probiotics consist of defined populations of specific microbial strains – typically bacteria or yeast – delivered in controlled doses. In contrast, FMT involves the transfer of processed fecal material from a rigorously screened healthy donor, containing a complex and diverse microbial community, to the recipient.

In terms of safety, both approaches are generally well tolerated in children. The most commonly reported side effects of probiotics are mild gastrointestinal symptoms such as bloating and gas.^50,51^ However, rare cases of probiotic-associated bacteremia and fungemia have been documented, predominantly in immunocompromised or critically-ill children.^41,42^ Nevertheless, most of these cases have been successfully treated with appropriate antimicrobial therapy. Similarly, most adverse events from FMT are mild and transient, including abdominal discomfort and diarrhea. Serious adverse events, such as infections caused by donor-derived pathogens, are rare but possible (0.9%), and has led to one death.^49^ Notably, long-term follow-up studies in pediatric populations have not reported increased risks of autoimmune, metabolic, or neoplastic diseases attributable to FMT.^52^ These small but very tangible risks underscore the need for caution when selecting and administering probiotics and FMT to vulnerable patients.

Probiotics have demonstrated efficacy in reducing late-onset sepsis and mortality among preterm and VLBW neonates based on data from more than 100 trials with over 25,000 infants.^43^ Meta-analyses indicate a 25–60% relative risk reduction in late-onset sepsis with probiotic supplementation.^43^ However, inconsistencies across studies remain, with some reporting no effect likely due to heterogeneity in probiotic strains, dosages, and study designs.^39,40^ The most commonly used probiotic species are of the genera *Lactobacillus* and *Bifidobacterium*, with the occasional yeast *Saccharomyces boulardii*. Well-studied strains with documented pediatric safety and efficacy include *Lactobacillus rhamnosus* GG, *Bifidobacterium breve*, *Bifidobacterium infantis*, and *Lactobacillus acidophilus*.^41^ These probiotics are available in various formulations – single strain or multi-strain blends, capsules, powders, and liquids – with typical doses ranging from 1 to 5 billion colony-forming units per day.

In contrast, clinical application of FMT in pediatric infections remains limited. Between 2002 and 2022, only 681 children across 18 clinical trials were reported to have received FMT, primarily for recurrent CDI and refractory diarrhea.^49^ There are no published clinical studies evaluating FMT for neonatal sepsis, and evidence for its broader use in pediatric infections remains sparse – relying largely on extrapolations from adult and preclinical research.^46,53^ As a safety measure, FMT donor samples are collected from rigorously screened healthy individuals to minimize the risk of pathogen transmission. However, the microbial composition of FMT material varies between donors, as there is currently no universally defined “healthy” microbiome signature.^54^ In addition, recent studies suggest that the success of FMT depends not only on donor microbial composition but also on the compatibility between donor and recipient microbiota – highlighting the importance of personalized matching, similar in principle to organ transplantation.^55^

In clinical practice, probiotics are often used adjunctively during antibiotic treatment.^51^ However, antibiotics can adversely affect probiotic viability and colonization by directly killing probiotic strains, potentially diminishing their therapeutic effects. To mitigate this, probiotics are generally given several hours apart from antibiotics to minimize direct antagonism.^56^ Conversely, FMT protocols typically require discontinuation of antibiotics prior to transplantation to enhance donor microbiota engraftment and restoration of the recipient’s gut flora.^57^ This requirement poses a clinical challenge in septic neonates who need ongoing antibiotic therapy, highlighting a critical gap in knowledge regarding the optimal combination between microbial-based and antibiotic treatments.

Thus, a significant unmet need remains in detailed characterization of the composition, pharmacokinetics and pharmacodynamics of probiotics and FMT products. Unlike conventional drugs, these biologics must survive gastric transit, colonize and interact dynamically with the host microbiota to exert their effects. Their therapeutic activity depends on multiple factors, including formulation, dosage, delivery route, and the baseline composition of the host microbiome. Addressing these gaps is essential to improve consistency in clinical outcomes and to develop optimized, evidence-based protocols for integrating microbial therapies into pediatric care.

## 4. Infections in pediatric cancer patients

In addition to neonates and infants, pediatric cancer patients represent another group at elevated risk of infection. This increased susceptibility is particularly pronounced in children due to their developing immune systems. The presence of hematological cancers, such as leukemia and lymphoma, in pediatric populations further exacerbates this risk for infection by directly compromising the immune response capabilities. Research indicates that pediatric patients with hematologic malignancies are threefold more likely to experience infections compared to those with solid tumors and that these infections account for 60% of the morbidity in pediatric cancer.^58–61^ Despite the recent stabilization in childhood cancer rates, the emergence of early-onset cancers presents a concerning trend, potentially increasing the number of young patients diagnosed with cancer in the near future.^62–64^ As such, there is a critical need for strategies to mitigate, manage, or predict infections in pediatric oncology.

Several mechanisms contribute to the heightened risk of infection in cancer patients. Hematological malignancies directly impair lymphocyte’s function, increasing susceptibility to opportunistic infections. Cancer treatments, including chemotherapy and hematopoietic stem cell transplantation (HSCT), compromise mucosal barriers (intestinal, oral and dermal), facilitating bacterial translocation to otherwise sterile sites. Bacteria such as *Enterococcus*, *Staphylococcus aureus*, and *Escherichia coli* are often implicated in these infections.^65^ These therapies also exert immunosuppressive effects, reducing the activity of immune effector cells like natural killer (NK) and T cells.^66^ Further, cancer treatments themselves induce dysbiosis, favoring the colonization of pathobionts, a situation exacerbated by weakened immunity and compromised mucosal barriers. Consequently, infections in cancer patients can manifest in various forms, including bloodstream infections (BSIs), oral mucositis, and gastrointestinal infections. It should be noted that not all BSIs lead to sepsis, as only 30% of sepsis cases are attributed to BSI.^67,68^ This complexity underscores the importance of multifaceted approaches in managing infection risks in oncology, particularly in pediatric cases.

### 4.1. Gut microbiome and cancer treatment

Pediatric cancers, particularly acute lymphoblastic leukemia (ALL), manifest unique microbiome signatures in the gut and oral cavity compared to healthy counterparts, suggesting a potential interplay between microbiota composition and disease pathogenesis or treatment response.^69–72^ Although the gut microbiome diversity in ALL patients was not significantly different to that of healthy children, structure of the microbial community, i.e., the proportions of the membership, shows significant differences between ALL and health controls, indicating individualized microbial compositions across the patient cohort.^71,73^ This was manifest even at the phylum level, with the increase in the proportion of *Bacteroidetes* and a reduction in *Firmicutes* in ALL patients.^71,72,74^ However, the exact compositional variations and their implications in disease progression or treatment response warranted further investigation.

Chemotherapeutic interventions and HSCT have profound effects on the gut microbiota, often leading to dysbiosis and a significant reduction in microbial diversity, a phenomenon more pronounced than impact from antibiotics.^69,70,73–75^ Specifically, chemotherapy for ALL patients not only substantially reduced the gut microbial diversity but also resulted in compositional structural shifts characterized by an increased proportion of the genus *Ruminococcus* and a diminished presence of the genus *Bacteroides*.^71,76,77^ This difference may influence a host’s metabolic processes and immune function. Patients undergoing HSCT face a more severe and persistent form of intestinal dysbiosis compared with those receiving chemotherapy alone (Table 1).^70^ This was evidenced by a sustained lower Shannon Diversity index over a 210-day period, coupled with a marked proliferation of *Proteobacteria*, a phylum that contains many pathogenic and proinflammatory species. Such long-lasting microbial imbalance may result from the HSCT procedure, including the extensive use of parenteral nutrition and broad-spectrum antibiotics during the post-transplant period. Notably, similar shifts are observed in adult recipients, where significant alterations to the microbiome remain detectable even six months after HSCT.^78^ Cancer interventions, while necessary, may inadvertently suppress beneficial bacterial populations and promote the dominance of opportunistic pathogens, potentially leading to complications such as infections and graft-versus-host disease.

### 4.2. Gut microbiome and infection prediction

In pediatric oncology, emerging evidence underscores the predictive capacity of the microbiome for infection-related complications. Notably, microbial signatures, far from being mere reflections of a disease state, have been strongly correlated with, and can potentially forecast, infectious events in pediatric patients with cancer. In a pivotal study involving 199 children newly diagnosed with ALL, Hakim et al elucidated that specific microbial taxa could predict the onset of neutropenic fever and chemotherapy-associated diarrhea (Table 1).^74^ Intriguingly, a gut microbiome profile at diagnosis exhibiting more than 0.01% *Proteobacteria* was associated with a 67% risk of febrile neutropenia development. Similarly, the gut microbiome composition at the commencement of chemotherapy was indicative of infection risk; an *Enterococcaceae* proportion > 30% was linked to an increased likelihood of febrile neutropenia and diarrhea, whereas a *Streptococcaceae* proportion > 30% was associated with a heightened risk of diarrhea.

A case-control study further substantiated these findings, revealing that pediatric ALL patients who later developed BSI had a baseline intestinal microbiome markedly deficient in *Bacteroidetes* (Table 1).^61^ Notably, fecal *Proteobacteria* were detectable up to 15 days before the clinical onset of BSI.^76^ Additionally, oral mucositis, a severe consequence of oral mucosa inflammation post-cancer therapy, demonstrated a significant correlation with the presence of fungal *Candida spp* (Table 1).^77^

These findings not only underscore the prognostic potential of the microbiome in pediatric oncology but also reveal the pathogenic roles of specific microbes. The observed association between the depletion of *Bacteroidetes* and therapeutic intervention or severe infection was notable given its generally positive influence on host health through immunomodulation and detoxification mechanisms.^61^ Conversely, the detection of pathobionts such as *Proteobacteria*, *Enterococcaceae*, and *Streptococcaceae* aligned with the onset of febrile neutropenia, diarrhea, and BSI, likely due to their pathogenic potential. These microbes are detected non-invasively in fecal samples prior to the manifestation of clinical symptoms, which offer a critical window for early intervention, potentially averting severe complications and improving patient outcomes.

### 4.3. Gut microbiome and infection prevention

Interventions targeting the microbiota have shown potential in reducing infection incidence in pediatric cancer patients. One approach, as demonstrated by Severyn et al, involved “gut decontamination” using oral vancomycin-polymyxin B prior to HSCT in ALL patients (Table 1).^79^ Although this intervention did not significantly alter the microbial diversity, Graft vs host Disease (GvHD) incidence, or immune profiles, it markedly reduced the incidence of BSIs. The study observed BSIs in 5 non-treated patients compared to only 1 in the treated group. Gut decontamination appeared to inhibit pathogens known to cause BSIs, such as *Klebsiella*, *Leclercia*, and *Staphylococcus*, suggesting its effectiveness in reducing BSI risk. However, the application of antibiotics in controlling infections must be approached cautiously to ensure efficacy and minimize the emergence of multi-drug resistant organisms. Prophylactic antibiotic usage during cancer treatments may lead to reduced antibiotic effectiveness. For instance, the use of ciprofloxacin prophylaxis followed by ceftazidime to treat febrile neutropenia in pediatric cancer patients resulted in an increased minimum inhibitory concentration (MIC) for ceftazidime, requiring higher doses for effective antimicrobial activity (Table 1).^80^ An alternative strategy using non-antibiotic agents with antimicrobial properties had also proven effective. For example, the addition of oral xylitol wipes to standard oral care for both pediatric and adult HSCT recipients (from chemotherapy to 28 days post-HSCT) significantly reduced oral infections, including gingivitis, oral plaques, and ulceration (Table 1).^81^ More importantly, this approach substantially decreased BSIs from oral pathogens in the xylitol group compared to controls.

Administering probiotics is another strategic approach to positively influence the gut microbiota. Initial studies confirmed the safety of probiotics for children undergoing HSCT. A study involving *Lactobacillus plantarum* administered from day −8 to + 14 of the HSCT procedure to 30 pediatric patients demonstrated a safety profile without incidents of *Lactobacillus* bacteremia or adverse events (Table 1).^82^ Furthermore, a trial by Wada et al involving *Bifidobacterium breve* strain Yakult administered thrice daily for six weeks to 42 pediatric cancer patients undergoing chemotherapy showed promising results (Table 1).^83^ This regimen led to reduced febrile neutropenia and less usage of parenteral antibiotics (0.5 vs 0.95 episodes and 3.22 vs 6.90 days, respectively). The improved clinical outcome was coupled with the reduced abundance of facultative anaerobes like *Enterococcus*, suggesting that *B. breve* could restrain the proliferation of potentially pathogenic species. A commercial synbiotic product, LactoCare, combining *Lactobacillus* and prebiotics, was tested on 113 children with ALL (Table 1).^84^ Post one week of administration, the synbiotic group reported a lower incidence of chemotherapy-induced diarrhea (3.7% vs. 11.5%) and a significant drop in constipation rates compared to the placebo group (14–20% less). This indicated potential clinical benefits by enhancing the intestinal environment in immunocompromised patients. However, the selection of probiotics is currently limited to commercially available products, as introducing new microbial strains in immunocompromised hosts carries inherent risks.

FMT is another intervention, especially for treating refractory CDI in pediatric cancer patients. Case reports have described FMT as generally well tolerated in this high-risk population; however, treatment outcomes were variable – only 2 of 4 patients achieved symptom resolution after a single FMT, and one patient required up to six FMTs, with recurrent CDI.^85,86^ Beyond CDI, FMT is being explored for its potential to restore gut microbiota balance disrupted by cancer therapies and antibiotic, which may reduce the incidence of GvHD and infections in HSCT patients.^87,88^ However, no clinical trials have evaluated the safety and efficacy of FMT for infection treatment in pediatric cancer patients outside of CDI. Experts still cautioned against routine FMT use in immunocompromised pediatric cancer patients due to significant infection risks associated with the procedure.^89^ Until more robust pediatric data are available, FMT in this population should be considered only in highly selected cases, preferably within clinical trial settings.

Together, these findings underscore the necessity for exploring diverse interventions via the gut microbiota to reduce infection risk in pediatric cancer patients, while also emphasizing the need for careful consideration and additional research to better optimize the use of antibiotics, probiotics and FMT.

## 5. Conclusion

The gut microbiota plays an important role in protecting neonates and children from infection, supporting immune development, and maintaining intestinal health. Infections such as sepsis and complications from cancer treatment are closely linked to disruptions in the microbiota, especially after antibiotics and chemotherapy. This review highlights growing evidence that microbiota-targeted strategies – including targeted antibiotic, probiotics and FMT – may help restore microbial balance and reduce infection risks in pediatric populations.

However, the current evidence has several limitations. First, the number of high-quality, large-scale clinical trials in pediatric populations remains limited. Most existing studies are observational, single-center, or focus narrowly on select conditions such as CDI, limiting generalizability. Second, the heterogeneity in microbial interventions – including strain selection, dosing, timing, and combination with antibiotics – makes it difficult to establish standardized protocols. Third, there is a lack of long-term safety data, for both probiotics and FMT in immunocompromised children, where risks of pathogen transmission remain a concern.

Future studies should focus on rigorous randomized clinical trials that assess the safety and efficacy of microbiota-targeted interventions across diverse pediatric populations. Longitudinal studies examining microbiota recovery following different treatment modalities are also needed to guide timing and personalization of interventions. In addition, greater integration of microbiome profiling into clinical practice may enable earlier detection of dysbiosis and risk stratification for infection. Ultimately, a deeper understanding of host – microbiota – pathogen interactions will pave the way for precision microbiome-based strategies to improve infection outcomes and reduce antimicrobial resistance in pediatric care.

## Data Availability

This systematic review is based on previously published studies and publicly available data. All data analyzed in this study were obtained from peer-reviewed articles, clinical trials, and scientific databases as cited in the manuscript. No new data were generated or collected for this review. Further inquiries can be directed to the corresponding author.
